# Exposure to Beta-(1,3)-D-Glucan in House Dust at Age 7–10 Is Associated with Airway Hyperresponsiveness and Atopic Asthma by Age 11–14

**DOI:** 10.1371/journal.pone.0098878

**Published:** 2014-06-06

**Authors:** Dharini Maheswaran, Yiye Zeng, Moira Chan-Yeung, James Scott, Alvaro Osornio-Vargas, Allan B. Becker, Anita L. Kozyrskyj

**Affiliations:** 1 Faculty of Medicine, Nursing, and Health Sciences, Monash University, Melbourne, Victoria, Australia; 2 Department of Pediatrics, Faculty of Medicine and Dentistry, University of Alberta, Edmonton, Alberta, Canada; 3 Department of Medicine, University of British Columbia, Vancouver, British Columbia, Canada; 4 Dalla Lana School of Public Health, University of Toronto, Toronto, Ontario, Canada,; 5 Department of Pediatrics and Child Health, Faculty of Medicine, University of Manitoba, Winnipeg, Manitoba, Canada; 6 School of Public Health, University of Alberta, Edmonton, Alberta, Canada; Cincinnati Children's Hospital Medical Center, United States of America

## Abstract

**Background:**

Mould exposure has been linked to childhood asthma and bronchial hyper-responsiveness. Few studies have assessed beta-(1,3)-d-glucan (beta-glucan), a significant fungal cell wall constituent, in relation to asthma in adolescence.

**Objective:**

To determine whether house dust-derived beta-glucan exposure at age 7–10 is associated with the development and persistence of atopic and non-atopic asthma, and bronchial hyper-responsiveness (BHR) by age 11–14.

**Methods:**

Dust samples were collected from the 1995 Study of Asthma, Genes, and Environment (SAGE) birth cohort. This cohort was derived from Manitoba provincial healthcare administrative records of children high and low risk for asthma. Samples were collected from the homes of 422 children at age 7–10 and analyzed using beta-glucan and endotoxin-specific Limulus Amoebocyte Lysate assays. Asthma, atopy, and BHR status of each child were also assessed at ages 7–10 and 11–14.

**Results:**

At age 7–10, beta-glucan dust levels in the home were associated with persistent atopic asthma at age 11–14 (OR 1.79 for each unit increase in levels, 95% CI 1.14–2.81), independent of endotoxin exposure, and *Alternaria* or *Cladosporium* sensitization. The likelihood of BHR almost doubled with unit increases in dust beta-glucan in asthmatic children. In children without asthma, exposure to high beta-glucan levels at age 7–10 also elevated risk for BHR in adolescence (OR 1.74, 95% CI 1.05–2.89). New-onset atopic asthma was twice more likely following high beta-glucan exposure in children without asthma but the association did not reach statistical significance. No associations were evident with concurrent asthma phenotype at age 7–10 or non-atopic asthma at age 11–14.

**Conclusion:**

These findings implicate home beta-glucan exposure at school-age as a risk factor for persistent atopic asthma and new-onset BHR. The higher prevalence of BHR in urban adolescents may be propagated by this home exposure.

## Introduction

Asthma is one of the most prevalent chronic respiratory disorders, affecting over 2 million individuals in Canada [1], 25 million in the United States [2] and 235 million persons worldwide [3]. Childhood asthma, in particular, places significant burden on families. The cost of childhood asthma to the Canadian healthcare system has been estimated at approximately 600 million dollars per year [4]. While asthma prevalence is leveling off in most age groups in Western countries [5], it is increasing among adolescents [6]. To date, childhood overweight and atopic sensitization have been identified as risk factors for adolescent asthma or bronchial hyper-responsiveness [7], [8]. Aside from environmental tobacco smoke [9], few studies have tested home environment exposures at school-age as putative risk factors for asthma development in adolescence [6].

Indoor mould has been self-reported to be present in an inordinately high percentage of homes of asthmatic children [10], and found to be associated with prevalent asthma [11]–[14] and severe bronchial hyper-responsiveness [15], [16] in cross-sectional studies of children and young adults. In a meta-analysis of 10 studies, numeric estimation of association places the greater likelihood of current asthma among persons living in homes with visible mould or dampness at 37%. A similar risk was reported for asthma development in 4 studies, although the summary odds ratio was not statistically significant [17]. In an update to this meta-analysis, Tischer et al [14] found prevalent asthma to be 49% higher among children with visible mould exposure. However, self-reported indoor mould has long been known to be subject to reporting bias [18]. Incorporating home inspection as a more objective measure, Pekkanen et al [19] found moisture damage to be related in a dose-dependent manner to asthma severity in children, as well as to asthma persistence and exacerbation. Although utilization of unbiased inspectors improves mould exposure assessment [20], this method remains susceptible to human error as well.

Beta-(1,3)-d-glucan (beta-glucan) is a glucose polymer present in fungal cell walls [21] which, due to its proposed immunomodulatory effects, is regarded as a biological response modifier [22], [23]. Though it is also found in certain plant species and bacteria [20], beta-glucan in dust is an accurate surrogate for indoor fungal exposure to yeasts, mushrooms and filamentous moulds, [23] representing the beta-glucan content of most indoor fungal species [24]. Beta-glucan has been found to be positively associated with peak expiratory flow (PEF) variability in asthmatic children [22], with respiratory symptoms in schoolchildren [25] and with bronchial hyper-responsiveness (BHR) in adults [26]. Alternatively, beta-glucan in house dust has been associated with reduced wheeze in infants and young children in some populations [27], [28]. In an uncontrolled trial of 6–12 year old children, subcutaneous administration of beta-glucan increased cytokine interleukin-10 levels, and reduced wheeze symptoms but not bronchodilator use [29]. Finally, although beta-glucan is expected to be a major biochemical component of most fungal biomass [23], it is associated with stronger immunomodulatory effects than observed mould damage [28], [30], [31]. This observation supports beta-glucan's established role in innate immune activation [23] and suggests differing mechanisms and/or independent risks from mould exposure.

In summary, home environment exposures, in particular to fungal beta-glucan, have been understudied as determinants of adolescent asthma. Using data from the population-based Study of Asthma, Genes, and Environment (SAGE) cohort, the primary aim of our investigation was to determine if exposure to beta-glucan in house dust at school-age was associated with the development and persistence of asthma and its phenotypes by the age of 11–14. Outcomes included the common phenotypes of atopic and non-atopic asthma, as well as BHR.

## Methods

### Ethics Statement

The SAGE study was approved by the University of Manitoba Human Research Ethics Board. Informed consent from parents and assent from their children were obtained at all recruitment stages.

### Study Population and Design

**Figure 1:**
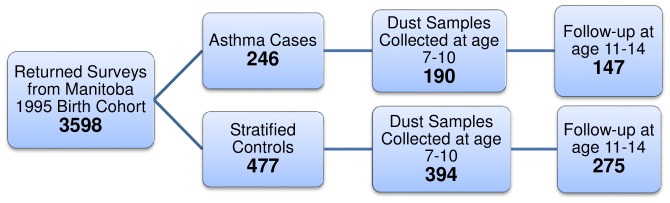
Flowchart of recruitment design. All parents of children from the 1995 Manitoba birth cohort who were alive and still resided in Manitoba were sent a short recruitment survey to which 3598 responded. These were grouped into cases and controls based on asthma status. Non-asthmatic controls were stratified according to urban or rural residence. Dust samples were collected from 190 and 394 cases and controls respectively. Of these, 77% of asthmatic children (n = 147) and 70% of control children (n = 275) were followed up for re-assessment by a pediatric allergist at age 11–14.

This was a planned, longitudinal follow-up of a nested case-control study of children in the 1995 SAGE birth cohort to study home environmental risk factors for asthma development in adolescence. This cohort was created from healthcare registry records of all children born in the province of Manitoba, Canada. Details of the study design for the SAGE cohort and nested case-control study have been published [32]. In brief, a short recruitment survey was sent to cohort households, including general health and home environment questions, as well as permission to be contacted. From the 3598 returned surveys (Figure 1) and following written consent from their parents, children aged 7–10 with and without asthma were recruited for the case-control study. This included all asthma cases (n = 246) and almost twice as many controls (n = 477), which had been randomly selected within urban-rural strata. Home dust samples were collected in 190 (77%) of homes of asthmatic children and in 394 (83%) of control homes. Seventy seven percent of asthmatic children (n = 147) and 70% of control children (n = 275) were seen at age 11–14 years (Figure 1). To test hypotheses on incidence and persistence, as others have [8], associations between dust beta-glucan and subsequent asthma and BHR were determined separately within each of the asthma and control arms.

### Study Variables

At ages 7–10 and ages 11–14, children were assessed by a pediatric allergist to diagnose asthma, according to Canadian Asthma Consensus guidelines [33] and skin tested for 16 common allergens (including tree pollen mix, weed pollen mix, ragweed, grass pollen mix, *Alternaria, Cladosporium, Penicillium*, house dust mites, cockroach, cat, dog, feathers and peanut). Atopic asthma was defined as a positive skin test for any allergen in the presence of diagnosed asthma; non-atopic asthma represented absence of sensitization to these allergens. BHR was determined using the Cockcroft method for the methacholine challenge test on the basis of a PC20 value of less than 8 mg/mL [34]. Overweight was determined as a BMI (body mass index) z-score of 1.04 (≥85^th^ percentile for age and gender) from the mean of 3 height and weight measurements using CDC growth charts [35]. At child age 7–10, parents completed detailed questionnaires on their home (eg. water damage, visible mould, pets, carpeting), their health (eg. family history of asthma, smoking) and their post-secondary educational background. A home inspection was conducted to collect a dust sample using the same portable vacuum cleaner for each home. A new cloth bag was attached for each collection and the vacuum was cleaned with an alcohol swab between homes. Samples were collected and combined from the child's mattress, bedroom floor, and most commonly-used room and frozen to −22°C.

Beta-glucan levels in collected dust samples were measured with the Glucatell assay (Associates of Cape Cod, East Falmouth, MA, USA), a modified Limulus Amebocyte Lysate (LAL) assay. Factor C, originally used for endotoxin detection, was replaced by Factor G, allowing for (1→3)-β-D-glucan detection and avoiding false positive results from cross detection. The Glucatell assay predominantly measures fungal glucans but can also detect glucans from plants sources [36]

A 5 mg extraction of each dust sample was diluted with 1 mL 0.3N Sodium Hydroxide (NaOH) and shaken at room temperature for one hour. The LAL and chromogenic substrate solutions were then added. After being placed on an incubating plate reader for 20 minutes at 37°C, the reaction was stopped by adding sodium nitrite, followed by ammonium sulfamate and N-1-Napthyl-ethylenediamine dihydrochloride. To quantify beta-glucan, the absorbance of the sample was measured with a spectrophotometer and compared to the absorbance of a standard curve. A log-transformed measure for beta-glucan levels was tested in prevalence models since beta-glucan was found to be log-normally distributed. To accommodate smaller sample size in incidence models, a binary format of the beta-glucan measure was implemented, using the median of 36.81 as the cut-off value. Log-transformed endotoxin levels were included in analyses to account for co-occurrence with beta-glucan in home dust [31].

### Statistical Analysis

Using ordinary logistic regression, the likelihood (odds ratios, ORs) for BHR, and prevalent and incident asthma and atopic asthma was determined for dust beta-glucan levels. Based on the adolescent asthma literature [7]–[9], as well as statistical significance of each variable when added to beta-glucan and endotoxin models, confounding factors such as water damage (ever reported), visible mould (reported by parent and seen by research assistant during the home visit), season (fall and winter versus spring and summer), urban/rural residence, in-home carpeting, atopic sensitization (any and specific for *Alternaria, Cladosporium, Penicillium*), family history of asthma, parental smoking, overweight and gender were tested in regression models. Confounding variables were retained in models according to statistical significance at a p<0.05 value and the “10% change in estimate rule,” whereby variables not found to be statistically significant but which changed the effect of beta-glucan by 10% or more were also included in the final model [37]. Hosmer-Lemeshow test was used to measure the Model Goodness-of-Fit. For incidence analyses, new-onset atopic asthma at mean age 12.5 was assessed by excluding children with asthma at age 7–10. All statistical analyses were performed by statistician and co-author, YZ, using the SAS (version 9.2) and SPSS statistical program for Windows (version 18.0). Based on odds ratios and underlying beta-coefficients for mold exposure from Behbod et al and Jo et al [38], [39] our study had sufficient power for atopic asthma (88%) and BHR (93%) in the prevalence analyses with log-transformed beta-glucan (n = 147), and for BHR (89%) but not for atopic asthma (54%) in the incidence analysis of high-low beta-glucan levels (n = 275) [40].

## Results

Data from 238 boys and 184 girls in the follow-up of the nested case-control study were available for analysis (Table 1). When children were 7–10 years old, 35% had asthma, 47% were atopic (22% were sensitized to *Alternaria*, 14% were sensitized to *Cladosporium*, 3.6% were sensitized to *Penicillium*) and 23% had a paternal history of asthma. The majority (94%) of parents had completed high school and almost one third were smokers. 57% of children lived in urban areas and still resided in their birth home. Any report of visible mould confirmed by a home visit was found in 50%, wall-to-wall carpeting in 51% and previous water damage in 46% of homes. Mean beta-glucan concentrations varied by season, from 36.58 mg/g in December-February and 53.90 mg/g in March-May to 63.08 mg/g in June-August and 79.38 mg/g in September-November. One quarter of homes contained beta-glucan levels in excess of 62.31 mg/g; more often these samples were taken in the fall/winter months (Table 1).

**Table 1:** Percentage Distribution of Confounding Factors in Relation to House Dust Beta-Glucan Exposure at Age 7–10 and Child Asthma at Age 11–14.

		Beta-Glucan Exposure a		Child (Atopic/Non-Atopic) Asthma		Child Atopic Asthma	
	No.	n (%)	p-value	n (%)	p-value	n (%)	p-value
**CHILD CHARACTERISTICS**							
**Gender**							
BoysGirls	238 184	55 (23.1) 50 (27.2)	NS	55 (29.9) 71 (29.8)	NS	34 (18.6) 55 (23.4)	NS
**Overweight, Age 7–10**							
YesNo	122 300	29 (25.3) 76 (23.8)	NS	36 (29.5) 90 (30.0)	NS	26 (21.5) 63 (21.2)	NS
**Asthma, Age 7–10**							
YesNo	147 275	66 (24.0) 39 (26.5)	NS	98 (66.7) 28 (10.2)	<.001	74 (50.7) 15 (5.5)	<.001
**Atopy**							
YesNo	199 221	48 (24.1) 57 (25.8)	NS	85 (42.7) 40 (18.1)	<.001	81 (41.1) 7 (3.2)	<.001
**Alternaria**							
YesNo	91 329	20 (22.0) 85 (25.8)	NS	44 (48.4) 81 (24.6)	<.001	44 (48.4) 44 (13.5)	<.001
**Cladosporium**							
YesNo	57 363	11 (19.3) 94 (25.8)	NS	30 (52.6) 95 (26.2)	<.001	30 (52.6) 58 (16.2)	<.001
**Penicillium**							
YesNo	15 405	3 (20.0) 102 (25.2)	NS	7 (46.7) 118 (29.1)	0.15	7 (46.7) 81 (20.2)	0.02
**Residence Location**							
UrbanRural/First Nation^b^	239 183	79 (33.1) 26 (14.2)	<.001	96 (40.2) 30 (16.4)	<.001	67 (28.4) 22 (12.1)	<.001
**Different Birth home**							
YesNo	239 183	63 (26.4) 42 (23.0)	NS	82 (34.3) 44 (24.0)	0.02	54(22.9) 35 (19.2)	NS
**Family History** c							
YesNo	95 320	30 (31.6) 73 (22.8)	0.08	29 (30.5) 95 (29.7)	NS	20 (21.3) 67 (21.1)	NS
**Parent's PS Education**							
YesNo	366 24	94 (25.7) 8 (33.3)	NS	112 (30.6) 8 (33.3)	NS	78 (21.5) 6 (26.1)	NS
**Smoking Parent** d							
YesNo	116 267	37 (31.9) 63 (23.6)	0.09	32 (27.6) 82 (30.7)	NS	23 (20.0) (22.0)	NS
**HOME CHARACTERISTICS**							
**Pets** e							
YesNo	354 54	84 (23.7) 16 (29.6)	NS	99 (28.0) 19 (35.2)	NS	65 (18.5) 16 (30.2)	0.05
**Carpet**							
YesNo	216 206	56 (25.9) 49 (23.8)	NS	63 (29.2) 63 (30.6)	NS	45 (21.0) 44 (21.6)	NS
**Water Damage** f							
YesNo	194 226	46 (23.7) 59 (26.1)	NS	49 (25.3) 75 (33.2)	0.08	38 (20.0) 50 (22.1)	NS
**Visible Mould** g							
YesNo	211 159	58 (27.5) 36 (22.6)	NS	66 (31.3) 49(30.8)	NS	42 (20.3) 38 (23.9)	NS
**Endotoxin**							
YesNo	102 302	34 (33.3) 69 (22.9)	0.04	31 (30.4) 89 (29.5)	NS	20 (20.0) 64 (21.3)	NS
**Season**							
Spring/SummerFall/Winter	292 113	66 (22.6) 37 (32.7)	0.04	87 (29.8) 34 (30.1)	NS	58 (19.9) 26 (23.6)	NS

Definition of abbreviations: PS – Post-Secondary, NS – Non-Significant (p-value >0.10).

a Beta-glucan levels in house dust.

b Manitoba First Nation community.

c Self-reported physician-diagnosed maternal or paternal asthma.

d Self-reported maternal or paternal smoking in the last 12 months.

e Presence of one or more pets in the home, frequent visitation of furry pets to the home, weekly contact with cats, dogs, farm, or other animals outside of the home.

f Self-reported water damage to the home since moving in.

g House inspector-reported mould or mildew seen on visible surfaces in the home within the last 12 months and self-reported mould found in the bathroom, bedroom, living room, or kitchen.

Thirty percent of children had asthma at age 11–14 and 21% had atopic asthma (Table 1). During the follow-up period, 10.2% children developed asthma and 27.8% developed bronchial hyper-responsiveness. Almost 6% were new cases of allergic-asthma and 4.4% were new cases of non-allergic asthma. One third of asthmatics were no longer asthmatic at age 11–14 years. Atopic asthma developed in 40% of children with atopy at age 7–10 and in half of children with sensitization to *Alternaria*. Children lost to follow-up significantly more often had parents who smoked (44%) but were less likely to be asthmatic (26%) or live in urban areas (45%); no others differences were found (Table 1). Separately, in the asthma and control arms, loss to follow-up was greatest among families living in rural areas (p<0.001) and families with parents who smoked (p<0.01).

Urban homes had the highest levels of beta-glucan compared with rural/First Nations (FN) homes (Table 1). The correlation between beta-glucan and endotoxin levels was weak (r = 0.14, p<0.0008), but high beta-glucan levels were found in homes with high endotoxin levels. Beta-glucan levels tended to be highest in homes of children with parental asthma or smoking. A higher percentage of children living in urban homes had asthma or atopic asthma at either age. Gender, child overweight, low socioeconomic status (as measured by parent education), in-home carpet and visible mould did not differ statistically by beta-glucan exposure or asthma development (Table 1).

I. Cross-sectional Analyses: Beta-Glucan Exposure and Asthma or BHR at Age 7–10

*Alternaria* sensitization at age 7–10 was strongly associated with current asthma (OR 2.82, 95% CI 1.85–4.29), atopic asthma (OR 7.10, 95% CI 4.51–11.2) and BHR (OR 2.26, 95% CI 1.40–3.65). Similarly, a positive skin prick test for *Cladosporium* (OR 6.61, 95% CI: 3.98–11.0) and *Penicillium* (OR 8.25, 95% CI: 3.25–20.9) were highly associated with atopic asthma. However, beta-glucan levels measured in house dust were not associated with asthma (adjusted OR 1.15, 95% CI 0.94–1.54), atopic asthma (adjusted OR 1.21, 95% CI 0.94–1.41), nonatopic asthma (adjusted OR 1.01, 95% CI: 0.76–1.35) or BHR (adjusted OR 1.01, 95% CI 0.83–1.22) in unadjusted models or models adjusted for endotoxin, and *Alternaria*, *Cladosporium* or *Penicillium* at this age.

### II. Longitudinal Analyses: Beta-Glucan Exposure at Age 7–10 and Asthma at Age 11–14

#### a) Prevalent asthma in children with previous asthma

Independent of household endotoxin levels, asthma prevalence at age 11–14 was 1.62 times more likely (95% CI 1.06–2.47) in children with existing asthma with each unit increase to dust beta-glucan levels (Table 2). This statistical association remained following adjustment for a positive skin prick test to *Alternaria* (OR 1.68, p<0.02) or *Cladosporium* (OR 1.64, p<0.02) or *Penicillium* (OR 1.62, p<0.03) at age 7–10. Asthma prevalence at age 11–14 was unrelated to *Penicillium* sensitization (OR 0.87, 95% CI:0.23–3.31). None of the other studied covariates (season, visible mould, urban residence, in-home carpet, parental smoking or asthma) affected this association and were not retained in models (Table 2). Although asthma at age 11–14 was statistically distributed by urban/rural residence (see Table 1), this association was no longer evident following stratification by existing asthma status.

**Table 2:** The Effect of Beta-Glucan Exposure at Age 7–10 on the Prevalence of Asthma, Atopic Asthma, and BHR in Children with Asthma.

Exposure Variable		Risk for Asthma		Risk for Atopic Asthma		Risk for BHR	
		OR (95% CI)	p-value^e^	OR (95% CI)	p-value^e^	OR (95% CI)	p-value^e^
**Model 1**	**Log(Beta-Glucan)** a	1.40 (0.94,2.09)	0.10	1.42 (0.96,2.09)	0.08	1.73 (1.12, 2.68)	0.01
**Model 2**	**Log(Beta-Glucan)** a	1.62 (1.06,2.47)	0.03	1.53 (1.02,2.30)	0.04	1.87 (1.19, 2.92)	0.006
	**Log (Endotoxin)** b	0.55 (0.32,0.94)	0.03	0.77 (0.48,1.25)	-	0.75 (0.45, 1.28)	-
**Model 3**	**Log(Beta-Glucan)** a	1.68 (1.09,2.59)	0.02	1.79 (1.14,2.81)	0.01	1.95 (1.23, 3.09)	0.004
	**Log (Endotoxin)** b	0.52 (0.30,0.91)	0.02	0.70 (0.41,1.20)	-	0.73 (0.42, 1.27)	-
	**Alternaria** c	2.14 (0.95,4.82)	0.07	5.89 (2.59,13.40)	<.0001	2.46 (1.02, 5.91)	0.04
**Model 4**	**Log(Beta-Glucan)** a	1.64 (1.07,2.52)	0.02	1.68 (1.08,2.60)	0.02	1.90 (1.21,2.98)	0.01
	**Log (Endotoxin)** b	0.56 (0.33,0.96)	0.03	0.81 (0.48,1.35)		0.77 (0.45,1.33)	-
	**Cladosporium** d	2.85 (1.06,7.70)	0.04	6.99 (2.60,18.75)	<.0001	2.86 (0.99,8.21)	0.05

Definition of abbreviations: CI – confidence interval; OR – odds ratio; BHR – Bronchial Hyper-Responsiveness.

a Beta-glucan levels in house dust.

b Endotoxin levels in house dust.

c *Alternaria* sensitization on skin prick test.

d *Cladosporium* sensitization on skin prick test.

e p-values >0.10 not reported.

#### b) Incident asthma in children without asthma

High beta-glucan levels (> median of 36.81) in house dust doubled the risk for new-onset asthma in adjusted models in children without pre-existing asthma, but statistical significance was not attained (Table 3, p<0.09). A statistical association with incident asthma was present for *Alternaria* but not *Cladosporium* or *Penicillium* sensitization. The association was unaffected by other study covariates.

**Table 3:** The Effect of Beta-Glucan Exposure at Age 7–10 on the Incidence of Asthma, Atopic Asthma, and BHR in Children without Asthma.

Exposure Variable		Risk for Asthma		Risk for Atopic Asthma		Risk for BHR	
		OR (95% CI)	p-value^e^	OR (95% CI)	p-value^e^	OR (95% CI)	p-value^e^
**Model 1**	**High Beta-Glucan** a	1.69 (0.76,3.75)	-	1.63 (0.57,4.72)	-	1.59 (0.98, 2.57)	0.06
**Model 2**	**High Beta-Glucan** a	2.09 (0.89,4.90)	0.09	2.81 (0.82,9.56)	0.10	1.80 (1.09, 2.97)	0.02
	**Log (Endotoxin)** b	0.95 (0.56,1.61)	-	0.45 (0.22,0.91)	0.03	0.79 (0.57, 1.11)	-
**Model 3**	**High Beta-Glucan** a	2.05 (0.87,4.81)	0.10	2.71 (0.78,9.42)	-	1.74 (1.05, 2.89)	0.03
	**Log (Endotoxin)** b	0.95 (0.56,1.62)	-	0.47 (0.23,0.96)	0.04	0.79 (0.56, 1.12)	-
	**Alternaria** c	1.38 (0.48,3.96)	-	3.47 (1.04,11.61)	0.04	2.23 (1.07, 4.63)	0.03
**Model 4**	**Log(Beta-Glucan)** a	2.10 (0.90,4.91)	0.09	2.73 (0.80,9.30)	-	1.76 (1.06.2.92)	0.03
	**Log (Endotoxin)** b	0.94 (0.55,1.61)	-	0.44 (0.21,0.90)	0.03	0.78 (0.55,1.10)	-
	**Cladosporium** d	0.39 (0.05,3.03)	-	0.85 (0.10,7.01)	-	2.42 (0.94,6.20)	0.07

Definition of abbreviations: CI – confidence interval; OR – odds ratio; BHR – Bronchial Hyper-Responsiveness.

a Beta-glucan levels in house dust.

b Endotoxin levels in house dust.

c *Alternaria* sensitization on skin prick test.

d *Cladosporium* sensitization on skin prick test.

e p-values >0.10 not reported.

### III. Longitudinal Analyses: Beta-Glucan Exposure at Age 7–10 and Atopic Asthma at Age 11–14

#### a) Prevalence asthma in children with asthma

Independent of endotoxin exposure, atopic asthma prevalence at age 11–14 was 1.5 times more likely (95% CI 1.02–2.30) in asthmatic children with each unit increase in exposure to beta-glucan (Table 2). Adjustment for *Alternaria* sensitization at age 7–10, which independently increased the risk for atopic asthma almost 6-fold (p<0.0001), strengthened the association between beta-glucan and atopic asthma prevalence (OR 1.79, 95% CI 1.14–2.81). Despite a stronger association between *Cladosporium* sensitization and subsequent atopic asthma, the association between beta-glucan and this asthma phenotype was unchanged. None of the other studied covariates affected this association (Table 2).

#### b) Incidence asthma in children without asthma

In children without previous asthma (atopic or non-atopic) at age 7–10, a statistical association between beta-glucan and atopic asthma at age 11–14 was not present (Table 3).

### IV. Longitudinal Analyses: Beta-Glucan Exposure at Age 7–10 and Non-atopic Asthma at Age 11–14

Beta-glucan levels in house dust were not found to be significantly related to non-atopic asthma at age 11–14 in crude or endotoxin-adjusted models (adjusted OR 1.01, 95% CI 0.60–1.69). Since no child with *Alternaria* or *Cladosporium* sensitization at age 7–10 developed nonatopic asthma, it was not possible to run models adjusted for these covariates.

### V. Longitudinal Analyses: Beta-Glucan Exposure at Age 7–10 and BHR at Age 11–14

Independent of endotoxin presence, unit increases to beta-glucan in house dust at age 7–10 were associated with a 2-fold increased likelihood of prevalent BHR at age 11–14 in children with existing asthma (OR 1.87, 95% CI 1.19–2.92, Table 2). *Alternaria* sensitization, an independent risk factor for BHR, did not alter the strength of this association (p<0.004, Table 2). In the absence of pre-existing asthma at age 7–10, exposure to high beta-glucan levels was also found to predict BHR independent of endotoxin levels. Specifically, the risk for BHR at age 11–14 was almost doubled in children without asthma (OR 1.80, 95% CI 1.09–2.97, Table 3). This likelihood was minimally affected following adjustment for *Alternaria* and *Cladosporium* (Table 3), or *Penicillium* (data not shown) sensitization.

### VI. Endotoxin Exposure at Age 7–10, Asthma Phenotype and BHR at Age 11–14

Independent of beta-glucan levels, exposure to endotoxin in house dust at age 7–10 reduced the risk for new-onset atopic asthma by one half (OR 0.47, 95% CI 0.23–0.96, Table 3). In children with pre-existing asthma, unit increases in endotoxin levels also halved the risk for persistent asthma (Table 2); the same non-statistical trend was evident for atopic asthma and BHR. Although statistical significance was not achieved, endotoxin levels were inversely associated with prevalent nonatopic asthma (OR 0.52, 95% CI: 0.26–1.05) but positively associated with incident nonatopic asthma (OR 1.50, 95% CI: 0.73–3.05). Adjustment for dust endotoxin levels frequently strengthened associations between beta-glucan, and asthma or BHR.

## Discussion

This community-based follow-up assessment of 422 Canadian children revealed that beta-glucan exposure in the home at age 7–10 was associated with asthma that persisted into adolescence. This risk for persistence was 1.68 times higher (95% CI 1.09–2.59) for each log unit increase in beta-glucan levels. Specifically, it was driven by the association with atopic asthma (adjusted OR 1.79, 95% CI 1.14–2.81) since beta-glucan exposure was not associated with non-atopic asthma. A 2-fold association was found for new-onset asthma following high beta-glucan exposure levels in children without asthma at age 7–10 but it was not statistically significant. Cross-sectionally at age 7–10, beta-glucan exposure was unrelated to asthma phenotype. Though sensitization to any allergen at age 6 is a stronger determinant of unremitting asthma [8], [41] this is the first study to report an association between beta-glucan in homes of school-age children and persistence of asthma into adolescence.

There are several additional important findings in this study. Beta-glucan in house dust at school age doubled the risk for subsequent BHR among children with asthma. BHR has been observed in asthmatic children exposed to high indoor beta-glucan levels in previous studies [22], [25], but a significant association between beta-glucan exposure and subsequent BHR in the absence of asthma is an original contribution of this study. Thus, our findings suggest that beta-glucan exposure can cause de novo BHR, as well as greater BHR in children with existing asthma. The association between indoor mould exposure and BHR has been shown to be unrelated to asthma and atopy previously, [42] suggesting a mechanism unique from that of atopic asthma. Not well-studied, the onset of puberty is a period of tissue plasticity and environmental exposures at this time may affect lung function [43]. Beta-glucans are known to have an effect on innate immunity through genetically predetermined pattern recognition receptors (PRRs) that bind to carbohydrates, lipids and proteins of foreign micro-organisms such as beta-glucan [44]–[46]. This binding of beta-glucan to PRRs on monocytes, macrophages and neutrophils [47] is crucial for its stimulation of immune activity [48]. In particular, beta-glucan is a powerful inducer of cytokines IL-6, IL-10, and IL-12 and thus a potent disruptor of normal immune responses [49]. This immunostimulatory effect may cause hypersensitivity, explaining the significant association between beta-glucan exposure and subsequent BHR in the absence of asthma.

Beta-glucan levels were not obviously higher in homes with visible mould, self-reported by the parent and observed during a home visit, and visible mould was not statistically significant in our models. Poor correlation between self-reported visible mould or fungal spores, and beta-glucan levels has been reported by others [27], [28], [50] Since airborne beta-glucan has been found to be inversely correlated with visible mould [51], lack of statistical modification by visible mould in our study suggests that beta-glucan in dust is a risk factor for asthma and BHR through its inhalation potential [50]. House dust mite has also been associated with allergic airway disease through beta-glucan dependent pathways [52], [53]. Season also did not affect model results despite a lower prevalence of high beta-glucan levels in study homes during the winter, as others have reported [50]. However, it is the aerosolized form of beta-glucan that induces inflammation in animal models [54] and no variations in winter-summer concentrations of aerosolized beta-glucan have been observed by others [50]. It was equally important to test the effect of urban-rural residence in models because beta-glucan levels were highest in urban homes and a greater proportion of children lost to follow-up were from rural areas. Higher beta-glucan in urban homes may be a function of a preference for carpeting [22], [55] and/or a higher weight of settled dust in urban homes compared with rural homes [31]. This adjustment did not change the beta-glucan association with asthma or BHR.

As others have found, atopic sensitization at age 7–10, in particular to *Alternaria* [41], was a strong risk factor for BHR or atopic asthma among children with and without concurrent asthma [8]. *Alternaria* is not a significant source of beta-glucan but co-exists with fungi that produce beta-glucan in house dust [24]; it minimally reduced the effect size for atopic asthma or BHR in our study. The independence of beta-glucan's and *Alternaria's* association with asthma and BHR suggest differing immunomodulatory pathways for each. Iossifova et al postulate that a low beta-glucan yet high allergenic protein content accounts for *Alternaria's* high allergic potential [24]. On the other hand, despite being a significant contributor to beta-glucan in house dust, [38] the beta-glucan association was also independent of *Cladosporium* sensitization in our study. Since sensitization to *Cladosporium* in Manitoba children is almost as common as for *Alternaria* [56] *Cladosporium* in outdoor air may also be a source of sensitization, as reported in the Behbod et al study [38].

The major strength of this study was temporal assessment between beta-glucan exposure at school age and subsequent adolescent asthma in a large number of children with and without asthma, as tested in incidence analyses and prevalence models. No association between beta-glucan and concurrent asthma at age 7–10 was observed, further providing evidence for its role in asthma development and persistence in adolescence. Since high beta-glucan levels co-existed with high endotoxin levels, all analyses were adjusted for endotoxin levels to parcel out the effect of beta-glucan. This adjustment often strengthened the association between beta-glucan and asthma or BHR. Found by others to have protective effects on asthma, wheeze [57], [58], and allergy [59] in young children, endotoxin exposure at school age halved the risk for new-onset atopic asthma. In our study, it also appeared to protect against the persistence of asthma into adolescence.

Despite its many strengths, our study was limited in the following ways. It had no additional measures of environmental exposures such as air pollution or beta-glucan in school dust [60]. We do not anticipate bias from the former omission because Manitoba has the second lowest air pollution index in Canada and air pollution plays a small causal role in asthma development [61], [62]. Personal smoking by adolescents was not measured in this study and the findings were not affected by parent smoking, although beta-glucan is also found in cigarettes [63]. Others have not found parent or adolescent smoking to be associated with adolescent asthma [41]. Children living in First Nations communities were grouped with rural children because of the small sample size (n = 31). Asthma prevalence is lower in both rural and First Nations children in Manitoba, so we did not anticipate a bias from this grouping. For economic reasons, dust samples from the living room and bedroom areas were combined at storage. Though within-home variance of beta-glucan measurements has been found to be small [64] this may not be applicable to all climates and populations. Our method for beta glucan detection [36] may also detect plant glucans and only one dust sample was collected over the 4 year assessment, which may not detect mould beta-glucan exposure changes. While Heinrich et al [65] concluded that beta-glucan levels in a single-point dust sample were representative of exposure for at least a one year period, this may not be true for all populations. Lastly, our incidence analysis was under-powered; the trend for new-onset asthma that we observed may become statistically significant in a larger sample.

## Conclusion

Our follow-up study of children with and without a genetic risk for asthma, found a risk for atopic asthma and BHR in adolescent children previously exposed to beta-glucan. Associations were found for exposure at age 7–10, indicating that beta-glucan in house dust is an important source of sensitization, even later in childhood. Replication of our findings in other populations is required. In the interim, allergen avoidance methods such as regular vacuuming and dry steam cleaning carpets can effectively reduce indoor dust [66] and beta-glucan levels [55]. These recommended practices for the management of childhood asthma and allergies may have benefit in preventing asthma persistence and development in adolescence. Since beta-glucan levels in our study were only slightly lower in the homes of children with existing asthma, and higher in the homes of parents who smoked or had a history of asthma, these recommendations may warrant reinforcement to prevent childhood asthma and allergen sensitivity.
